# ADVANCING FAMILY CAREGIVER HEALTH AS A KEY PUBLIC HEALTH PRIORITY

**DOI:** 10.1093/geroni/igae098.0198

**Published:** 2024-12-31

**Authors:** Courtney Polenick

**Affiliations:** University of Michigan, Ann Arbor, Michigan, United States

## Abstract

The Health and Aging Policy Fellowship (HAPF) offers exposure to the development and implementation of health policies at both the state and national levels. As a family caregiving researcher and the first Ralph C. Wilson, Jr. Foundation HAPF Caregiving Policy Track Fellow, my HAPF placements included the Office of U.S. Congresswoman Debbie Dingell and the National Alliance for Caregiving (NAC). These placements provided various opportunities to learn more about translating research findings into policies and actions that improve services and supports for older adults and their family caregivers. Based on extensive research showing that caregiving can lead to negative health effects, it is critical to consider family caregiver health as an important public health priority. My placement with the Office of Congresswoman Dingell focused on advancing legislation to enhance home and community-based services (HCBS), including supports for family caregivers. My placement with NAC focused on building upon their public health framework and further promoting family caregiver health as a key public health issue. We synthesized common priorities within existing public health frameworks that are applicable to family caregiving across the lifespan. We also identified upstream intervention and system change approaches for implementing parts of the 2022 National Strategy to Support Family Caregivers to advance family caregiver health within the public health landscape.

